# Kinematic Analysis of a Six-Degrees-of-Freedom Model Based on ISB Recommendation: A Repeatability Analysis and Comparison with Conventional Gait Model

**DOI:** 10.1155/2015/503713

**Published:** 2015-01-29

**Authors:** Magdalena Żuk, Celina Pezowicz

**Affiliations:** Department of Biomedical Engineering, Mechatronics and Theory of Mechanisms, Faculty of Mechanical Engineering, Wrocław University of Technology, Łukasiewicza 7/9, 50-371 Wrocław, Poland

## Abstract

*Objective*. The purpose of the present work was to assess the validity of a six-degrees-of-freedom gait analysis model based on the ISB recommendation on definitions of joint coordinate systems (ISB 6DOF) through a quantitative comparison with the Helen Hays model (HH) and repeatability assessment. *Methods*. Four healthy subjects were analysed with both marker sets: an HH marker set and four marker clusters in ISB 6DOF. A navigated pointer was used to indicate the anatomical landmark position in the cluster reference system according to the ISB recommendation. Three gait cycles were selected from the data collected simultaneously for the two marker sets. *Results*. Two protocols showed good intertrial repeatability, which apart from pelvic rotation did not exceed 2°. The greatest differences between protocols were observed in the transverse plane as well as for knee angles. Knee internal/external rotation revealed the lowest subject-to-subject and interprotocol repeatability and inconsistent patterns for both protocols. Knee range of movement in transverse plane was overestimated for the HH set (the mean is 34°), which could indicate the cross-talk effect. *Conclusions*. The ISB 6DOF anatomically based protocol enabled full 3D kinematic description of joints according to the current standard with clinically acceptable intertrial repeatability and minimal equipment requirements.

## 1. Introduction

Kinematic analysis of the human gait is an instrumented measurement allowing for a quantitative description of movement patterns, which provides useful data for clinical practice and biomechanical research. Gait analysis is primarily descriptive and its interpretation (especially clinical findings) is based largely on comparing patterns of movement to normative data [1]. However, kinematic analysis is limited by simplified biomechanical models determined by the marker sets. Current protocols of gait analysis differ considerably and, hence, the obtained results differ, too [2, 3]. Reliable comparisons of gait patterns, definition of standards, and the need to report kinematic variables in clinical terminology require anatomically based protocols for gait analysis [4].

The International Society of Biomechanics (ISB) proposed a general reporting standard for joint kinematics based on the Joint Coordinate System defined by anatomical landmarks [5]. On the other hand, Conventional Gait Model (CGM) and its variations (referred to as Helen Hayes, Newington, Davis III et al. [1, 6, 7]), which are mainly used for clinical purposes, are not fully anatomically based. In these models, markers are placed both on anatomical landmarks and wands, therefore the anatomical planes of the thigh and shank are defined by positioning wands [6]. Another limitation of commonly used protocols is a constraint of the ankle-foot complex with two degrees of freedom instead of a 3D description of motion [6, 7]. These limitations are partially explained by the fact that data acquisition techniques were developed for low resolution motion capture systems with a reduced number of cameras, which imposed the use of a few spaced markers [8]. Modern optical systems, capable of accurately measuring the positions of the markers with high frequency, as well as the number of their possible locations, are sufficiently advanced to not introduce significant errors to the gait analysis or limit the biomechanical model [1]. The current and inevitable limitation of an optical system is the need to maintain “line of sight” between a marker and the camera, which makes it difficult to track markers placed on medial sides of lower limbs during walking. Some anatomical landmarks that define coordinate frames according to the current ISB recommendation [5] are medially located; therefore, markers placed on these landmarks could be obscured by the opposite limb.

The use of a cluster of markers on each segment of the lower limb (6DOF marker set) [2] and the “calibrated anatomical systems technique” (CAST) [11, 12] allow to track any anatomical landmarks on the selected segments. A cluster consists of at least three noncollinear markers attached on a rigid base (rigid triad of markers) to a segment or directly to the skin [13]. Such marker set enables tracking of each segment independently, allowing 6DOF at each joint (rotational and translational) [2].

The purpose of the present work was to assess the validity of a six-degrees-of-freedom model based on the ISB recommendation on definitions of the joint coordinate system (ISB 6DOF). This will be accomplished through a comparative study of a proposed marker set and a modified Helen Hays-Davis protocol (HH) [6, 7] as the Conventional Gait Model. A quantitative comparison of kinematic measurement and intertrial variability for the two protocols was achieved by analysing the same gait acquisition in four healthy subjects. The variability was calculated according to the recommended method [10, 14].

A recent study evaluated performance of an anatomically based protocol [4], its repeatability for gait analysis in adult subjects [9], and its comparison to other current protocols [3]. Another study evaluated the six-degrees-of-freedom marker set using clusters [2]. However, both protocols are not fully consistent with the ISB recommendation.

## 2. Method

Four able-bodied subjects without a walking disability (two females and two males) were analyzed (aged 23 ± 2 years, weight 69.0 ± 17.2 kg, height 1.72 ± 0.15 m). All participants provided written informed consent before participation.

**Figure 1:**
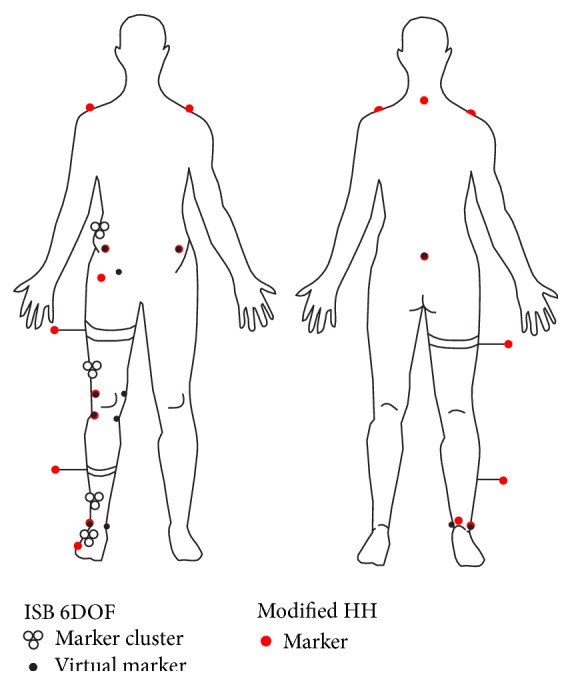
Marker locations for ISB 6DOF and HH sets. ISB 6DOF: triads of hollow circles—technical marker clusters, black, small dots—virtual markers; HH—red, big dots.

Two marker sets: ISB 6DOF and a modified Helen Hayes (HH) were applied concurrently to track motion of the right lower limb (Figure 1). One assessor indicated the marker position and the virtual marker position in each subject and both marker sets.

Twenty-two photoreflexive markers were placed on wands and anatomical landmarks according to a modified HH procedure (used BTS MAS model) [6, 7, 15]. Trajectories of the photoreflexive marker were recorded using the BTS Smart E (BTS Bioengineering, Milan, Italy) motion analysis system containing 6 digital infrared cameras. The obtained data were processed using BTS Smart Analyzer.

**Figure 2:**
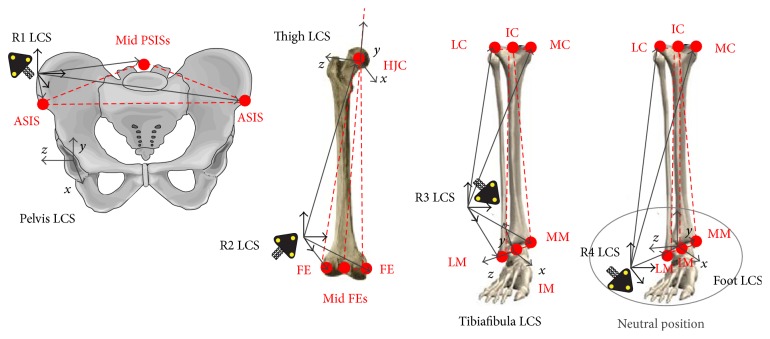
Joint Coordinate System definition according to ISB recommendation [5] based on anatomical landmarks measured with respect to the appropriate cluster of markers.

In ISB 6DOF, clusters of active markers (infrared LED) were located on the pelvis, thigh, shank, and foot. Each cluster consisted of three noncollinear active markers (infrared LED) attached on a rigid base (Optotrak Smart Marker Rigid Body, NDI, Canada) [16]. Positions of anatomical landmarks (tracked as virtual markers) with respect to the appropriate cluster (rigid triad of technical markers) were measured using the tracked pointer (equipped with markers) during a static trial (similarly as in [12]). While the dynamic trial, the instantaneous anatomical landmark positions were calculated on the basis of the position vector registered during static trial and the current Marker Rigid Body's transformation matrix. Anatomical reference frames were defined by selected bony landmarks according to the ISB recommendation (Figure 2) [5]. In particular, the foot coordinate system was defined by shank anatomical landmarks in a neutral position with respect to the foot technical frame, which is consistent with the recommendation. The hip joint centre was determined using Davis III et al.'s regression equation [7]. The three Cardan angles were used to describe the joint action of flexion/extension, adduction/abduction, and internal/external rotation [17]. Pelvic angles were defined as rotation angles between the pelvis coordinate system and the position sensor coordinate system (laboratory coordinate system).

Active marker cluster (for ISB 6DOF) trajectories were collected by one position sensor of the motion capture system (Optotrak Certus, NDI, Canada) with three embedded infrared cameras. Data acquisition and joint angle calculation were performed using software developed by the author. Furthermore, the data were processed using Matlab, including filtering of marker trajectories with a 4th order low-pass Butterworth filter (cut-off 6 Hz [18]) and joint angle normalisation to 101 points per cycle. The measurements from both systems were conducted simultaneously.

The subjects walked barefoot at a preferred pace. Three gait cycles for each subject were selected on the basis of good quality of marker trajectories for both marker sets. Two different kinematic descriptions, obtained for both protocols, were compared.

**Figure 3:**
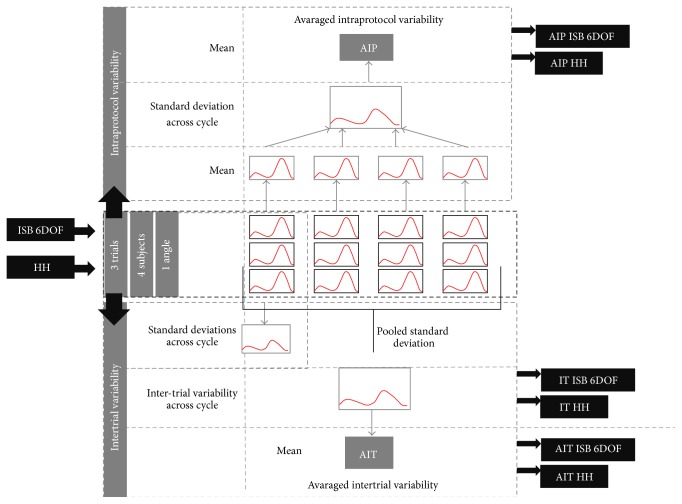
Scheme of the data processing procedure. Four healthy subjects were examined using two different protocols (ISB 6DOF, HH), three trials were selected. Averaged intraprotocol variability (AIP) and averaged intertrial variability (AIT) were calculated, intertrial variability across cycle (IT) was plotted for both protocols.

The mean value and the standard deviations of the 12 rotations were calculated for each sample of the gait cycle (from the three gait cycles), in both protocols, in four subjects. For visual comparison, joint angle curves were plotted for one representative subject (mean of three cycles) and for four subjects (averaged across mean curves of each subject) with ±1 SD confidence bands. Intertrial variability was calculated according to the recommended method [10, 14] and plotted for visual comparison of patterns for the two marker sets. Average inter-trial variability (AIT) were calculated for both marker sets and compared to the corresponding values from recent papers [13, 14]. Averaged intraprotocol variability (AIP) was defined as mean standard deviation over all subjects averaged across the gait cycle. Procedure of data processing for averaged intraprotocol variability, average intertrial variability, and intertrial variability across the gait cycle was presented in the flow chart (Figure 3). Interprotocol variability was calculated as the mean absolute variability (MAV), that is, absolute value of the difference between two angles (ISB 6DOF and HH) along frames averaged over all samples of cycle, calculated for each subject, and then averaged [3, 19].

## 3. Results

Joint rotations calculated from the two protocols are in good agreement with corresponding data obtained with the same (in the case of HH [15]) or similar protocol (in the case of ISB 6DOF [4, 12]).

**Figure 4:**
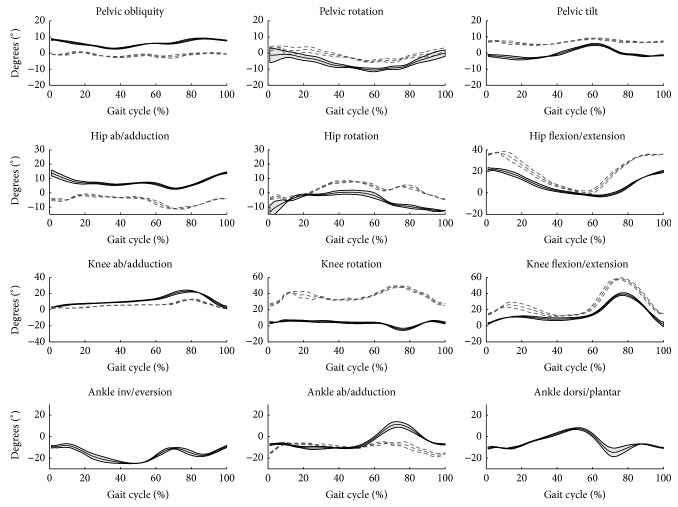
Kinematic variables as calculated by the two protocols, averaged across three cycles of one representative subject. HH—grey dashed line, grey thick line = ± SD, ISB 6DOF—black solid line, black thin lines = ± SD.

**Figure 5:**
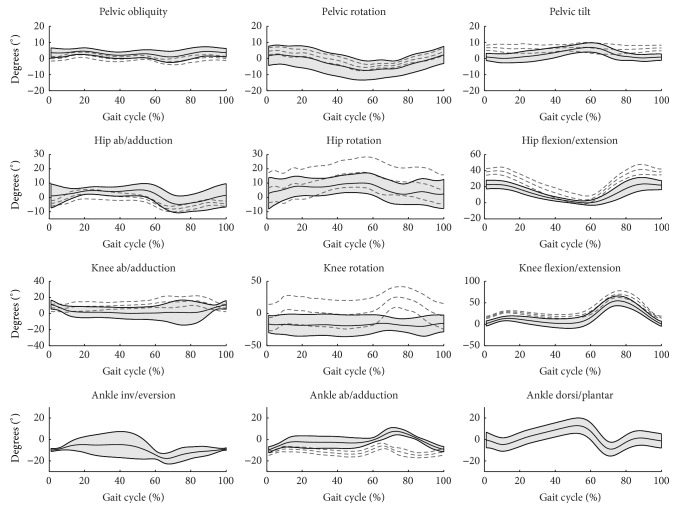
Kinematic variables as calculated by the two protocols, averaged across mean curves of the four subjects. HH—grey dashed line, grey thick line = ± SD, ISB 6DOF—black solid line, grey band = ± SD.

The highest consistency of general patterns of joint angle for both protocols is observed for the sagittal plane and the lowest for the transverse plane, especially for knee rotation (Figures 4 and 5). Judging from the MAV indicators (Table 1), the highest agreement among joints is for pelvis angles, where anatomical definitions for both protocols are the same, and the lowest for knee angles, where differences in anatomical definitions are greatest differences for knee angles in the frontal and coronal planes were observed both in the patterns of angles and the ranges of motion. The mean ranges of motion for knee angles are 11° (ISB 6DOF) and 34° (HH) for internal/external rotation and 22° (ISB 6DOF) and 11° (HH) for abduction/adduction.

**Figure 6:**
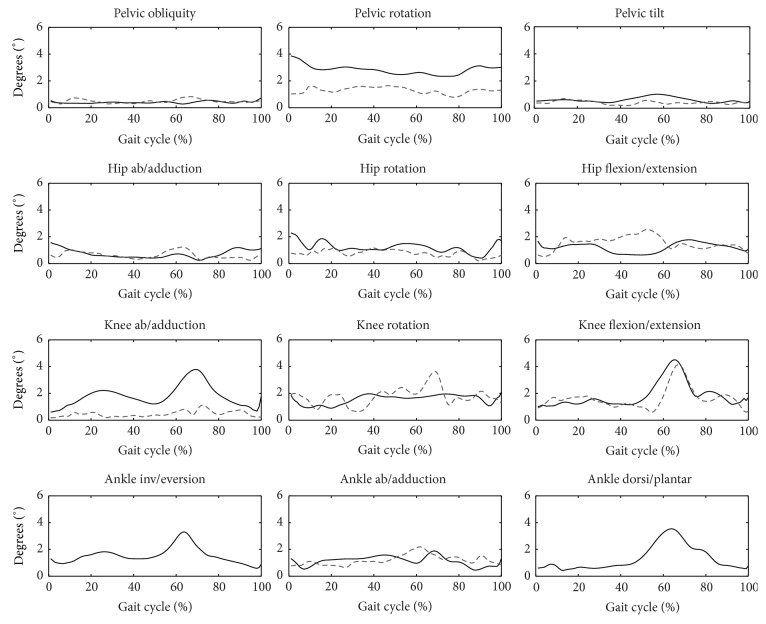
Patterns of intertrial variability across all samples of the gait cycle, average for four subject. HH—grey dashed line, ISB 6DOF—black solid line.

Intertrial variability is low for both protocols and similar to the corresponding data from previous work (Table 1). The highest mean intertrial variability is observed for knee angles and the lowest for pelvis angles for both protocols. The most repeatable rotation within the same subject is pelvic obliquity (0.3°) for the ISB 6DOF protocol and knee abduction/adduction (0.3°) for HH, and the least is knee abduction/adduction (3.2°) for ISB 6DOF and knee rotation for HH (2.9°). Intertrial repeatability for knee and ankle angles clearly depends on the phase of gait (Figure 6). The lowest repeatability is observed during the swing phase, in particular for knee flexion for both protocols. A decrease of repeatability during the swing phase occurs for knee abduction/adduction for the ISB 6DOF protocol (while it does not occur for HH) and this decrease occurs for knee rotation for HH (while it does not occur for ISB 6DOF). Intraprotocol variability is highest for knee internal/external rotation and for both protocols (Table 1) and likewise for interprotocol variability.

## 4. Discussion

### 4.1. Comparison of Methods

The two protocols differ in their marker sets as well as in the adopted biomechanical models.

Conventional protocols require attachment of reflective markers on the skin in the area of anatomical landmarks, and in the case of the ISB 6DOF protocol the clusters are attached to segments by bands and anatomical calibration is performed with the navigated pointer. Despite these differences, the times of patient preparation for both marker sets are comparable. Both methods are affected by soft tissue artefacts (STA). For the HH protocol, the position of each single marker suffers from various errors associated with movement of the underplaying soft tissues, in contrast to marker sets with clusters, where errors in the position of virtual markers for the selected segment are equal and arise from displacement of the cluster relative to the bone. STA for cluster can be minimized by appropriate cluster positioning [13, 20]. An arrangement of marker clusters enables motion tracking by a single optical position sensor (consisting of at least two cameras) and defining of virtual markers beyond the line of sight of the position sensor. By adding additional anatomical calibration, a larger number of virtual markers (freely placed within the segment) can be tracked. This solution minimizes the required equipment and thus it could enhance the availability of gait analysis in both research and clinical application.

The Cardan angular convention is applied in both methods to calculate rotation angles for coordinate systems of two adjacent segments. However, definitions of the coordinate system vary considerably. In the HH model, axes are defined by markers placed on anatomical markers but also by wands, which are manually adjusted to define the frontal plane. In the case of ISB 6DOF, anatomical axes are fully defined by anatomical landmarks. Davis predictive method was applied to the femur head in both protocols; therefore, the effect of the hip joint centre (HJC) localization method is eliminated in this study. Application of a different hip HJC localization method would alter the estimated HJC position (by as much as several centimetres) and the resulting knee and hip angle in the sagittal and frontal plane [21, 22] as well as kinetic variables [8, 23]. Recently, various methods have been proposed and validated [24, 25] with proven better accuracy than Davis's regression equation. Therefore, more accurate methods for hip joint centre determination should be considered in anatomically based protocols for gait analysis.

### 4.2. Comparison of Results

The main differences in joint angle patterns for both protocols are due to different anatomical definitions, which is particularly prominent for an out-of-sagittal plane. Previous papers also confirm similar differences in the results for various protocols [2, 3].

Most joint rotations measured by the ISB 6DOF protocol are in good agreement with these from the literature [4, 12]. However, the range of motion (ROM) for the pelvic tilt appears to be somewhat high for ISB 6DOF. The calculated simultaneous hip flexion is lower than for HH, while ROMs for other knee angles are similar. It may indicate that soft tissue artefacts (STA) consist of slight movement of a pelvis cluster occurring during hip flexion at the beginning of the swing phase. This type of artefact is not associated with significantly reduced repeatability. Previous studies claimed that tracking a cluster of markers mounted to the pelvis is not different than conventional marker sets with single markers placed over a bony landmark [3, 12]. However, the cluster attachment method should be noted in the future.

For both protocols, the main reasons for differences in knee joint angle patterns are the anatomical definition inconsistency and soft tissue artefacts, which are known to have the greatest impact on knee joint angles [4]. The HH ROM in the transverse plane is larger than ISB 6DOF and seems less realistic, while coronal ISB 6DOF ROM is larger than HH. As the repeatability of knee flexion decreases, there is a simultaneous decrease of repeatability of knee internal/external rotation and knee abduction for, respectively, HH and ISB 6DOF. Knee joints rotations are known to be affected by the cross-talk effect [26]. It seems that HH knee internal/external rotation and ISB 6DOF knee adduction/abduction are affected by this to varying degrees (HH more than ISB 6DOF), which obviously alters other knee angles. Less cross-talk effect was observed for ISB 6DOF, which may indicate greater consistency with real joint rotation axes. The obtained results also indicate that the cross-talk effect affects both nonsagittal angles and the course of repeatability of the above during the gait cycle. HH knee internal/external rotation can also suffer from the effect of the anteroposterior motion of the greater trochanter and lateral epicondyle markers [4].

Placement of a cluster of markers on the foot enables a three-dimensional foot tracking (in contrast to conventional tracking with 1DOF for ankle motion) and definition of the foot reference frame to the neutral configuration of the ankle joint complex according to the current recommendation [5]. The rotation of the ankle joint complex is consistent with previous research [27] despite distinct definitions.

### 4.3. Comparison of Variability

Overall, there are no significant differences in the average repeatability for both protocols. It is possible to achieve very high repeatability for both protocols (even 0.3°). Average intertrial variability does not exceed 2°, except for ISB 6DOF pelvic rotation, which is directly related to the definition and caused due to altered gait direction. According to a previous paper [14], the results are acceptable in most common clinical applications. However, evaluation of the ISB 6DOF protocol suggests that intersession and interassessor repeatability should be examined in the future. Although there was no difference in average intertrial variability, the patterns of intertrial variability across the gait cycle differ for the two protocols. Despite relatively low average, intertrial variability may double during the swing gait, which should not be ignored (Figure 6). Intertrial variability is regarded as intrinsic variation of gait patterns [10, 14]; however, its patterns across the gait cycle differ for the two biomechanical models and marker sets. Intraprotocol variability measures the subject-to-subject variability, which is the highest for knee internal/external rotation in both protocols. On the other hand, interprotocol variability measures differences between the two protocols, which is also the highest for knee internal/external rotation.

## 5. Conclusion

Kinematic variables and their repeatability are best reported for Conventional Gait Models. The widespread use of conventional gait protocols allows worldwide clinical gait analysis data comparison. In contrast to protocols based on the ISB recommendation, whose implementation is not widespread or commercially available, ISB 6DOF marker set enables full 3D kinematic description of the lower limb with minimal required equipment (one position sensor composed of at least two cameras), which could enhance the availability of gait analysis in both research and clinical application. It is an anatomically based protocol, fully consistent with the current ISB recommendation [5], which appears to be useful in clinical application due to the need for a reliable intra- and intersubject comparison of kinematic gait variables reported in clinical terminology [4]. Additionally, this marker set allows for a functional method of determining the joint centres and axes of rotation, which has been attempted in previous papers and has given reasonable results [1, 21, 23, 28, 29]. Both protocols show similar, high intertrial repeatability, which is comparable to previous studies. The achieved repeatability is acceptable in clinical application; however, intersession and interassessor repeatability should be examined on a larger number of subjects. Satisfactory precision is still not coupled with high accuracy, which is essential in research as well as in clinical practise. Both models are not free of such limitations as soft tissue artefacts or the cross-talk effect. Another area of challenge is development of a protocol consistent with joint anatomy and functionality as well as its validation.

## Figures and Tables

**Table 1:** Average intertrial, intraprotocol variability and mean absolute variability over the gait cycle across four subjects. Corresponding values from Manca et al. [9] and Schwartz et al. [10]. ^*^Data estimated from figures provided.

Rotations [°]	Intertrial	Intertrial	Intraprotocol	Intraprotocol	Mean absolute variability (MAV)	Intertrial	Intertrial
	ISB 6DOF	HH	ISB 6DOF	HH	ISB 6DOF compared to HH		
	Present study	Present study	Present study	Present study	Present study	Manca et al. [9]	Schwartz [10]
Pelvis tilt	0.6	0.4	3.0	2.7	4.0	0.9	0.8^*^
Pelvis obliquity	0.4	0.5	2.8	1.7	3.1	1.4	0.5^*^
Pelvis rotation	2.8	1.3	5.8	1.8	4.2	1.7	1.0^*^
Hip flex/ext	1.2	1.5	5.4	5.3	12.6	1.8	1.2^*^
Hip abd/add	0.7	0.6	5.5	2.6	5.4	1.7	0.5^*^
Hip intr/extr	1.2	0.8	8.2	11.5	8.5	2.9	1.2^*^
Knee flex/ext	1.8	1.6	9.8	5.0	15.4	2.2	1.6
Knee var/valg	1.8	0.5	8.7	5.9	11.7	1.6	0.5^*^
Knee intr/extr	1.6	1.8	16.2	22.2	23.5	4.3	1.2^*^
Ankle dor/pla	1.3	—	7.2	—	—	2.0	1.3^*^
Ankle inv/ev	1.5	—	6.8	—	—	2.3	—
Ankle abd/add	1.1	1.2	4.0	2.4	10.3	2.8	1.7

## References

[1] Baker R. (2006). Gait analysis methods in rehabilitation. *Journal of NeuroEngineering and Rehabilitation*.

[2] Collins T. D., Ghoussayni S. N., Ewins D. J., Kent J. A. (2009). A six degrees-of-freedom marker set for gait analysis: repeatability and comparison with a modified Helen Hayes set. *Gait and Posture*.

[3] Ferrari A., Benedetti M. G., Pavan E. (2008). Quantitative comparison of five current protocols in gait analysis. *Gait and Posture*.

[4] Leardini A., Sawacha Z., Paolini G., Ingrosso S., Nativo R., Benedetti M. G. (2007). A new anatomically based protocol for gait analysis in children. *Gait and Posture*.

[5] Wu G., Siegler S., Allard P. (2002). ISB recommendation on definitions of joint coordinate system of various joints for the reporting of human joint motion—part I: ankle, hip, and spine. *Journal of Biomechanics*.

[6] Kadaba M. P., Ramakrishnan H. K., Wootten M. E. (1990). Measurement of lower extremity kinematics during level walking. *Journal of Orthopaedic Research*.

[7] Davis R. B., Õunpuu S., Tyburski D., Gage J. R. (1991). A gait analysis data collection and reduction technique. *Human Movement Science*.

[8] Della Croce U., Leardini A., Chiari L., Cappozzo A. (2005). Human movement analysis using stereophotogrammetry Part 4: assessment of anatomical landmark misplacement and its effects on joint kinematics. *Gait and Posture*.

[9] Manca M., Leardini A., Cavazza S. (2010). Repeatability of a new protocol for gait analysis in adult subjects. *Gait and Posture*.

[10] Schwartz M. H., Trost J. P., Wervey R. A. (2004). Measurement and management of errors in quantitative gait data. *Gait and Posture*.

[11] Cappozzo A., Catani F., Della Croce U., Leardini A. (1995). Position and orientation in space of bones during movement: anatomical frame definition and determination. *Clinical Biomechanics*.

[12] Benedetti M. G., Catani F., Leardini A., Pignotti E., Giannini S. (1998). Data management in gait analysis for clinical applications. *Clinical Biomechanics*.

[13] Cappozzo A., Cappello A., Croce U. D., Pensalfini F. (1997). Surface-marker cluster design criteria for 3-D bone movement reconstruction. *IEEE Transactions on Biomedical Engineering*.

[14] McGinley J. L., Baker R., Wolfe R., Morris M. E. (2009). The reliability of three-dimensional kinematic gait measurements: a systematic review. *Gait and Posture*.

[15] Pietraszewski B., Winiarski S., Jaroszczuk S. (2012). Three-dimensional human gait pattern—reference data for normal men. *Acta of Bioengineering and Biomechanics*.

[16] Żuk M., Krysztoforski K. Marker set for gait analysis and its implementation in custom software.

[17] Tupling S. J., Pierrynowski M. R. (1987). Use of cardan angles to locate rigid bodies in three-dimensional space. *Medical & Biological Engineering & Computing*.

[18] Winter D. A. (2009). *Biomechanics and Motor Control of Human Movement*.

[19] Palermo E., Rossi S., Marini F., Patanè F., Cappa P. (2014). Experimental evaluation of accuracy and repeatability of a novel body-to-sensor calibration procedure for inertial sensor-based gait analysis. *Measurement: Journal of the International Measurement Confederation*.

[20] Peters A., Sangeux M., Morris M. E., Baker R. (2009). Determination of the optimal locations of surface-mounted markers on the tibial segment. *Gait and Posture*.

[21] Żuk M., Świątek-Najwer E., Pezowicz C. (2014). Hip joint centre localization: evaluation of formal methods and effects on joint kinematics. *Information Technologies in Biomedicine*.

[22] Stagni R., Leardini A., Cappozzo A., Grazia Benedetti M., Cappello A. (2000). Effects of hip joint centre mislocation on gait analysis results. *Journal of Biomechanics*.

[23] Delp S. L., Maloney W. (1993). Effects of hip center location on the moment-generating capacity of the muscles. *Journal of Biomechanics*.

[24] Sangeux M., Peters A., Baker R. (2011). Hip joint centre localization: evaluation on normal subjects in the context of gait analysis. *Gait & Posture*.

[25] Leardini A., Cappozzo A., Catani F. (1999). Validation of a functional method for the estimation of hip joint centre location. *Journal of Biomechanics*.

[26] Cappozzo A., Della Croce U., Leardini A., Chiari L. (2005). Human movement analysis using stereophotogrammetry. Part 1. Theoretical background. *Gait and Posture*.

[27] Benedetti M. G., Manca M., Ferraresi G., Boschi M., Leardini A. (2011). A new protocol for 3D assessment of foot during gait: application on patients with equinovarus foot. *Clinical Biomechanics*.

[28] Schwartz M. H., Rozumalski A. (2005). A new method for estimating joint parameters from motion data. *Journal of Biomechanics*.

[29] Hicks J. L., Richards J. G. (2005). Clinical applicability of using spherical fitting to find hip joint centers. *Gait and Posture*.

